# Cholesterol metabolism regulator SREBP2 inhibits HBV replication via suppression of HBx nuclear translocation

**DOI:** 10.3389/fimmu.2024.1519639

**Published:** 2025-01-13

**Authors:** Fan Yang, Feng Hu, Hongxiao Song, Tie Li, Fengchao Xu, Jing Xu, Le Wang, Fei Wang, Yujia Zhu, Mian Huang, Yanli Gao, Min Rao, Haichun Ma, Guangyun Tan

**Affiliations:** ^1^ Department of Hepatology, Center for Pathogen Biology and Infectious Diseases, Institute of Translational Medicine, The First Hospital of Jilin University, Changchun, Jilin, China; ^2^ Department of Anesthesiology, The First Hospital, Jilin University, Changchun, Jilin, China; ^3^ Department of Gastroenterology, The First Hospital of Jilin University, Changchun, China; ^4^ Department of Rheumatology and Immunology, The First Hospital of Jilin University, Changchun, China; ^5^ Health Examination Center, The First Hospital of Jilin University, Changchun, China; ^6^ Department of Pediatrics, The First Hospital, Jilin University, Changchun, Jilin, China

**Keywords:** cholesterol metabolism, HBV replication, SREBP2, HBx, HBV - hepatitis B virus

## Abstract

The intricate link between cholesterol metabolism and host immune responses is well recognized, but the specific mechanisms by which cholesterol biosynthesis influences hepatitis B virus (HBV) replication remain unclear. In this study, we show that SREBP2, a key regulator of cholesterol metabolism, inhibits HBV replication by interacting directly with the HBx protein, thereby preventing its nuclear translocation. We also found that inhibiting the ER-to-Golgi transport of the SCAP-SREBP2 complex or blocking SREBP2 maturation significantly enhances HBV suppression. Notably, we demonstrate that the C-terminal domain (CTD) of SREBP2, rather than its N-terminal domain (NTD), mediates this inhibition by interacting with HBx and promoting its extracellular secretion, thus reducing nuclear HBx accumulation. These findings reveal a novel regulatory pathway that links cholesterol metabolism to HBV replication via SREBP2-mediated control of HBx localization. This insight provides a potential basis for new therapeutic strategies against HBV infection, addressing an important global health issue.

## Introduction

Chronic HBV infection affects over 240 million people worldwide, making it the leading viral liver disease and often leading to liver fibrosis, cirrhosis, and hepatocellular carcinoma (HCC) (1). Cirrhosis or HCC caused by HBV infection results in over 820,000 deaths annually, making it the second leading cause of cancer-related mortality globally (2). As a result, new therapeutic strategies are urgently needed to control HBV replication and transmission. The HBV genome is a 3.2 kb partially double-stranded circular DNA consisting of four open reading frames (ORFs) named pre-C/C, pre-S/S, P, and X (3). Among these, the X ORF encodes the HBx protein, a multifunctional regulator that plays a critical role in HBV pathogenesis and the development of HCC (4, 5). HBV RNA is typically transcribed from covalently closed circular DNA (cccDNA) in the nuclei of infected hepatocytes (6). HBx is a 154-amino acid protein with an N-terminal negative regulatory domain and a C-terminal trans-activating or co-activating domain. It directly or indirectly influences viral replication, proliferation, and pathogenesis. Notably, HBx promotes HBV RNA synthesis by facilitating the proteasome-mediated degradation of structural maintenance proteins chromosome 5/6 (Smc5/6), thereby enhancing HBV replication (7).

The sterol regulatory element-binding protein (SREBP) pathway regulates intracellular sterol homeostasis through a negative feedback mechanism, wherein high levels of cholesterol inhibit further cholesterol synthesis by preventing the activation of SREBP (8, 9). In mammals, there are two SREBP-encoding genes: SREBP1 and SREBP2. SREBP1 primarily regulates genes involved in fatty acid metabolism, while SREBP2 mainly regulates genes related to cholesterol metabolism, such as the low-density lipoprotein (LDL) receptor and cholesterol synthesis enzymes, including the rate-limiting enzyme 3-hydroxy-3-methylglutaryl-CoA reductase (HMGCR) (10, 11). SREBPs consist of an N-terminal transcription factor domain and a C-terminal regulatory domain, linked by a transmembrane hairpin loop. The C-terminal domain of SREBP binds to the C-terminal WD40 domain of SCAP (SREBP cleavage-activating protein) to form the SREBP-2-SCAP complex. When intracellular cholesterol is abundant, the SREBP-2-SCAP complex is anchored to the ER membrane through a sterol-dependent interaction between SCAP’s sterol-sensing domain (SSD) and Insig. This interaction responds more strongly to certain cholesterol derivatives, such as 25-hydroxycholesterol (25HC), than to cholesterol itself. When cholesterol is depleted, SCAP dissociates from Insig. The SREBP-2-SCAP complex is then transported to the Golgi by COPII vesicles, where it undergoes a two-step proteolytic cleavage mediated by site-1 protease (S1P) and site-2 protease (S2P). This releases the N-terminal transcription factor domain of SREBP, which is transported to the nucleus to activate the expression of genes involved in cholesterol synthesis and uptake (12, 13).

Research in various fields has shown that many physiological and pathophysiological processes are closely related to lipid metabolism (14). Cholesterol, an important lipid, is involved in multiple biological processes. It has been reported that cholesterol metabolism undergoes changes during HBV infection (15). Our previous study demonstrated that cholesterol hydroxylase CH25H can inhibit HBx nuclear translocation, leading to the suppression of HBV replication (16). The link between HBV replication and infection and cholesterol metabolism, however, remains to be fully understood. In this study, we focused on the essential viral regulatory protein HBx and the key transcriptional regulator of cholesterol biosynthesis, SREBP2. We found an interaction between SREBP2 and HBx, and that SREBP2 effectively inhibited HBV replication by reducing the nuclear entry of HBx.

## Method details

### Sample collection

This study enrolled 44 HBV-infected patients and 18 healthy controls (**Supplementary Tables S1, S2**). Venous blood samples (5 mL) were collected to extract serum and PBMCs. HBV DNA levels were measured using Roche’s COBAS TaqMan Kit, and liver function along with biochemical parameters was assessed using an automatic biochemical analyzer. These procedures were conducted in the Department of Hepatology, First Hospital of Jilin University, Changchun, China (NO.2023-552).

### Cell culture, plasmids, and reagents

HEK293T and HepG2 cells were cultured in DMEM supplemented with 10% inactivated fetal bovine serum, 100 IU/mL penicillin, and 100 mg/mL streptomycin at 37°C with 5% CO_2_. SREBP2, HBx, and their truncation constructs were generated by cloning the coding regions of the respective human genes into VR1012 vectors with Flag, HA, or GST tags, using the EASY-Uni seamless cloning kit (TransGen, Beijing, China). The primers used in this study are listed in **Supplementary Table S3**.

### Total RNA and HBV DNA extraction and quantitative PCR

Total RNA was extracted using TRIZOl (Invitrogen, San Diego, CA, USA) and reverse transcribed into cDNA with Superscript III Reverse Transcriptase (Invitrogen). HBV DNA was isolated from whole cell lysate according to the manufacturer’s instructions (TransGen, Beijing, China). GAPDH was used as an internal control; qPCR was performed as described elsewhere (17). The gene-specific primer sequences for qPCR are provided in **Supplementary Table S4**.

### Co-immunoprecipitation and Western blotting

Cells were lysed 24–48 h post-transfection in 50 mM Tris–HCl (pH 8.0), 150 mM NaCl, and 1% NP-40 with protease inhibitors (Roche, USA). For immunoprecipitation, lysates were incubated overnight with ANTI-FLAG^®^ M2 or ANTI-HA^®^ M2 Affinity Gel (Sigma, USA). Immunoblotting was performed as previously described (18). Briefly, cells were lysed in RIPA buffer with a protease/phosphatase inhibitor cocktail, incubated on ice for 30 min, with mixing every 10 min. Protein concentration was quantified using the Coomassie Plus™ Protein Assay reagent (Thermo Scientific, Rockford, IL, USA). Samples were separated by SDS-PAGE and transferred to PVDF membranes. After blocking in TBS with 0.1% Tween-20 and 5% skim milk, blots were probed with specific antibodies.

### Nuclear and cytoplasmic extraction

Nuclear and cytoplasmic fractions were prepared using the Nuclear and Cytoplasmic Protein Extraction Kit (Beyotime, China) and subjected to Western blotting with relevant antibodies.

### ELISA

HepG2 cells were transfected with SREBP2 expression plasmids together with pHBV1.2 ΔX or pHBV1.2-HBV expression plasmids. The supernatant was collected after 48 h for ELISA analysis to detect HBsAg and HBeAg levels (Kehua Shengwu, China).

### Immunofluorescence

HepG2 cells were transfected with Flag-HBx plasmids or co-transfected with HA-SREBP2 and truncation plasmids for 36 h, followed by fixation in acetone-methanol (1:1) at 37°C for 10 min. Cells were then washed with phosphate-buffered saline (PBS), blocked with 5% bovine serum albumin (BSA) in PBST for 1 h, and incubated with Flag (mouse) and HA (rabbit) antibodies at 37°C for 1 h. After washing in PBS, cells were incubated with goat anti-mouse IgG conjugated with FITC or Cy3- and FITC-conjugated IgG (Proteintech). Cells were washed with PBS and observed with a Nikon AXR confocal microscope.

### CRISPR/Cas9 knockout

HepG2 cells were seeded in 24-well plates; 16 h later, two plasmids—one expressing Cas9 with SREBP2 and another carrying a puromycin resistance gene (PL-GFP-IP)—were co-transfected into cells using ViaFect transfection reagent (Promega). At 36 h post-transfection, cells were either selected by adding puromycin (2 μg/mL) or collected for immunoblotting with specific SREBP2 antibodies. Two days post-transfection, single live cells were sorted into 96-well plates at 1 cell/well using a BD FACSAria. Immunoblotting was performed again to determine gene-editing efficiency, and DNA sequencing verified gene editing. sgRNA and DNA sequencing primers are listed in **Supplementary Table S5**.

### Cell proliferation assays

After treatment, cells were incubated with 10 μL of CCK-8 (Yeasen, China) for 3 h, and absorbance was measured at 450 nm. Each sample was measured in triplicate.

### Quantification and statistical analyses

Data analysis was performed with GraphPad Prism 10 (GraphPad Software, San Diego, CA) using a two-tailed unpaired t-test for between-group comparisons. Statistical significance was set at p < 0.05.

### Data and code availability

The published article includes all datasets generated or analyzed in this study. Additional datasets or code may be available upon request from the Lead Contact.

## Result

### SREBP2 inhibits HBV replication

**Figure 1:**
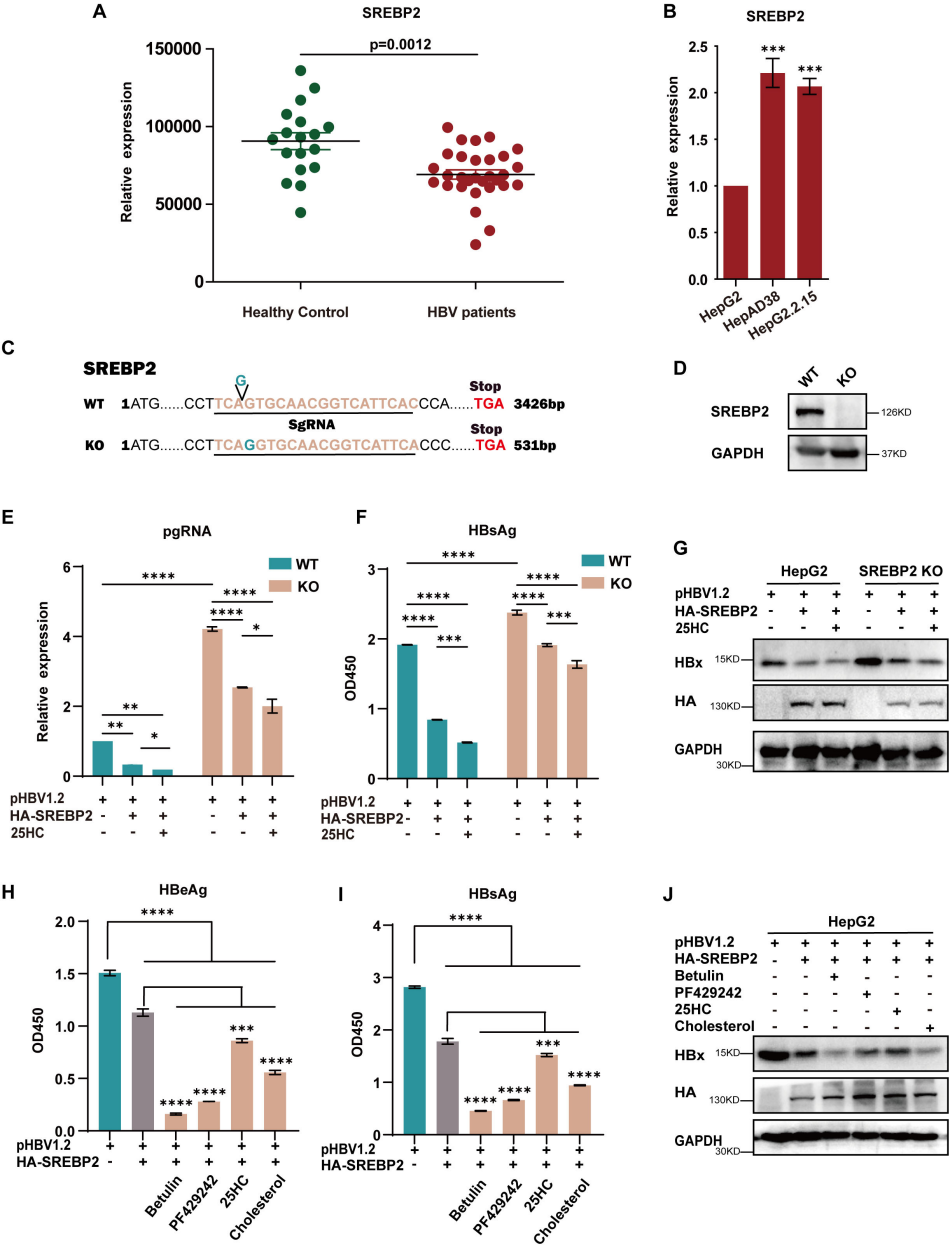
SREBP2 inhibits HBV replication. **(A)** SREBP2 mRNA expression was measured by qPCR in peripheral blood mononuclear cells (PBMCs) from healthy controls (n = 18) and HBV patients (n = 44). **(B)** SREBP2 mRNA levels were quantified by qPCR in HepG2, HepG2.2.15, and HepAD38 cells. **(C, D)** SREBP2 knockout (KO) in HepG2 cells was performed using CRISPR/Cas9 technology. The genomic DNA of wild-type (WT) and knockout (KO) cells was purified, and a 493 bp region surrounding the sgRNA target site was amplified and sequenced, the base G was inserted into the exon of SREBP2, resulting in a frameshift mutation that generated a premature stop codon at positions 529-531 bp. Western blot analysis was conducted to confirm SREBP2 knockout. **(E, F)** HepG2 cells were transfected with pHBV1.2 alone or co-transfected with HA-tagged SREBP2 expression plasmids, followed by treatment with 25-hydroxycholesterol (25-HC). After 48 hours, cells were harvested for qPCR analysis of pgRNA and ELISA for HBsAg secretion. **(G)** Immunoblotting was performed on cell lysates from **(E, F)** using antibodies against HA, HBx, and GAPDH. **(H, I)** HepG2 cells were transfected with pHBV1.2 alone or co-transfected with HA-tagged SREBP2 expression plasmids and treated with 10 mM betulinic acid, 50 mM PF-429242, 10 mM 25-HC, or 50 mM cholesterol. After 48 hours, supernatants were collected and analyzed by ELISA, with optical density (OD) measured at 450 nm. **(J)** Immunoblotting was performed on cell lysates from **(H, I)** using antibodies against HA, HBx, and GAPDH. *, p≤0.05; **, p≤0.01; ***, p≤0.001; ****, p≤0.0001.

SREBP2 is a key transcription factor involved in lipid metabolism. Given its critical role, we hypothesized that SREBP2 may play a significant role in the interplay between HBV replication and sterol regulation. To test this, we first investigated whether the expression of SREBP2 was modulated in response to HBV stimulation. Interestingly, we observed that SREBP2 expression was downregulated in peripheral blood mononuclear cells (PBMCs) from HBV-infected patients compared to healthy controls (**Figures 1A**). In contrast, SREBP2 was upregulated in HBV-replicating cell lines, including HepG2.2.15 and HepAD38 (**Figures 1B**). These seemingly contradictory findings prompted us to further explore the role of SREBP2 in HBV replication. We first used CRISPR-Cas9 technology to knock out the SREBP2 gene in HepG2 cells (**Figures 1C, D**). SREBP2 knockout led to increased levels of pgRNA, HBV DNA, HBsAg, and HBeAg, indicating that SREBP2 inhibits HBV replication. Conversely, overexpression of SREBP2 reduced these markers in both wild-type and knockout cells (**Figures 1E–G**, **Supplementary Figure S1A**). To further explore the mechanism, we treated cells with 25HC, an oxysterol that retains the SCAP-SREBP2 complex in the endoplasmic reticulum (19), and observed an enhanced inhibitory effect on HBV replication. Additionally, the use of betulinic acid, an inhibitor of SCAP-SREBP2 activation (20), along with cholesterol and PF-429242, further strengthened this suppression (**Figures 1H–J**). Importantly, no cytotoxic effects were observed after 24 hours of exposure to these inhibitors (**Supplementary Figure S1B**). Together, these results suggest that the inhibitory effect of SREBP2 on HBV replication is linked to its maturation, which involves the generation of its C-terminal domain (CTD) and N-terminal domain (NTD).

### SREBP2 interacts with the HBx protein

**Figure 2:**
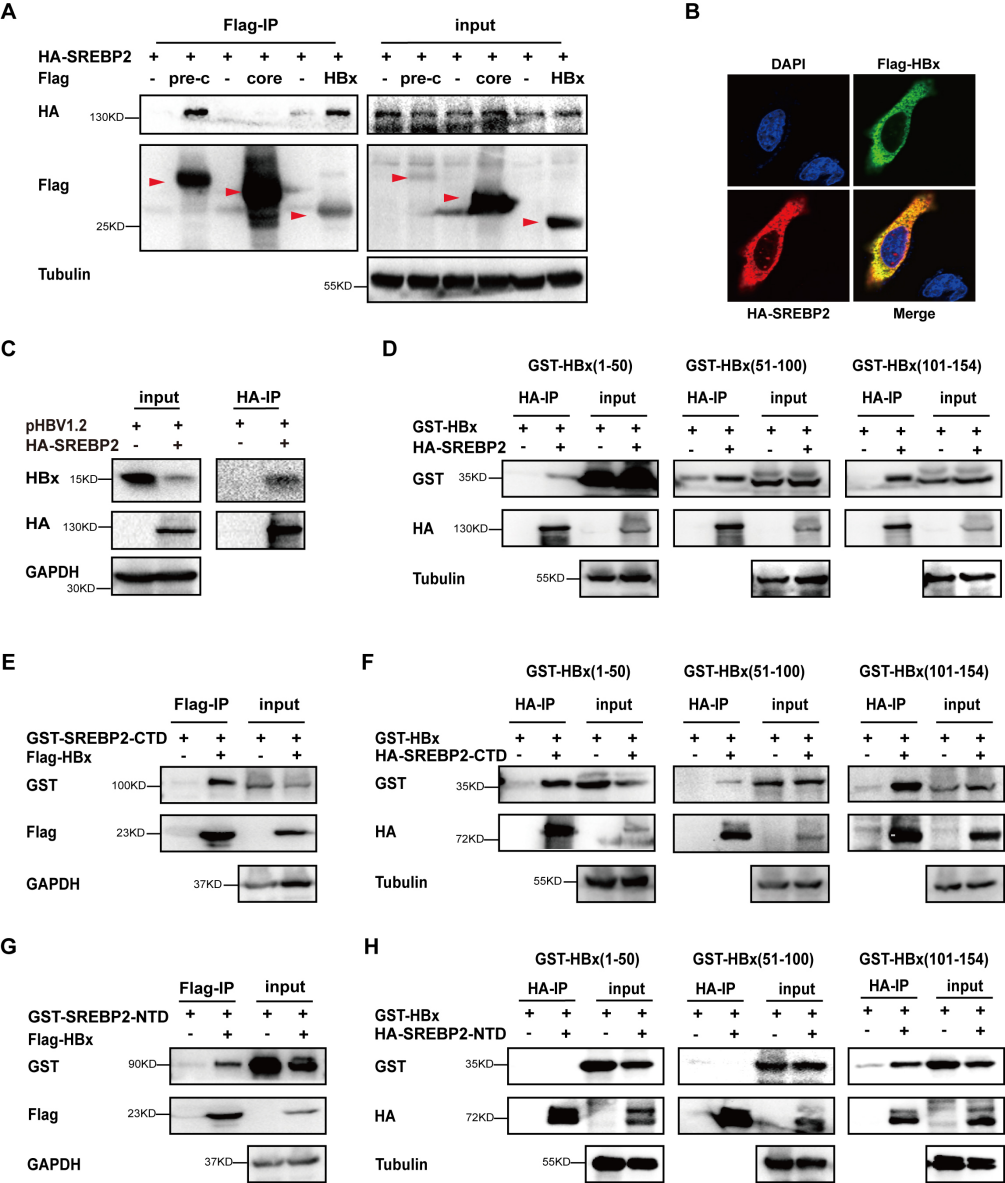
SREBP2 strongly interacts with HBx protein. **(A)** HepG2 cells were co-transfected with Flag-tagged HBV proteins (Core, preCore, and HBx) and HA-SREBP2 constructs. After 28 hours, co-immunoprecipitation (co-IP) was performed, and cell lysates and precipitates were analyzed by immunoblotting with anti-Flag, anti-HA, and anti-Tubulin antibodies. **(B)** HepG2 cells were co-transfected with Flag-HBx and HA-SREBP2. After 36 hours, cells were washed with PBS and analyzed by immunofluorescence using anti-HA and anti-Flag antibodies. **(C)** HepG2 cells were transfected with pHBV1.2 alone or co-transfected with HA-SREBP2. After 28 hours, cells were harvested for co-IP using anti-HA, anti-HBx, or anti-GAPDH antibodies. **(D)** Immunoprecipitation was performed in HepG2 cells co-transfected with GST-HBx truncations and HA-SREBP2 or empty vectors, followed by immunoblotting with anti-HA, anti-GST, or anti-Tubulin antibodies. **(E)** HepG2 cells were co-transfected with GST-SREBP2-CTD and Flag-HBX or empty vectors. After 28 hours, cells were subjected to co-IP with anti-Flag, anti-GST, or anti-GAPDH antibodies. **(F)** HepG2 cells were co-transfected with HA-SREBP2-CTD or empty vectors and GST-HBx truncations. After 28 hours, co-IP was performed, and cell lysates and precipitates were analyzed by immunoblotting with anti-HA, anti-GST, and anti-Tubulin antibodies. **(G)** Co-transfections were performed using GST-SREBP2-NTD and Flag-HBX or empty vectors in HepG2 cells, and co-IP analysis was conducted 28 hours post-transfection using anti-Flag, anti-GST, or anti-GAPDH antibodies. **(H)** HepG2 cells were co-transfected with HA-SREBP2-NTD or empty vectors and GST-HBx truncations. After 28 hours, co-IP was performed, and cell lysates and precipitates were analyzed by immunoblotting with anti-HA, anti-GST, and anti-Tubulin antibodies.

To better understand the mechanism by which SREBP2 inhibits HBV replication, we constructed plasmids expressing Pre-core, core, and HBx, which were co-transfected into HepG2 cells along with the HA-SREBP2 vector for co-immunoprecipitation (co-IP) assays. The results showed that both Pre-core and HBx interacted with SREBP2 (**Figure 2A**). Since HBx plays a critical role in HBV replication (20), we focused on investigating the interaction between SREBP2 and HBx. We performed immunofluorescence assays and observed co-localization of SREBP2 and HBx within the cells (**Figure 2B**). Overexpression of HA-SREBP2 in pHBV1.2-transfected HepG2 cells further confirmed the interaction of SREBP2 and HBx (**Figure 2C**). Next, to further delineate the interaction between SREBP2 and HBx, we generated truncations of HBx, including X1, X2, and X3, and constructed plasmids for SREBP2’s C-terminal domain (CTD) and N-terminal domain (NTD). These HBx truncation mutants were co-expressed with SREBP2 in HepG2 cells and subjected to co-IP assays. The results demonstrated that all three HBx truncations, X1, X2, and X3, interacted with SREBP2 (**Figure 2D**). Subsequently, we co-expressed the SREBP2-CTD and NTD domains with HBx in HepG2 cells and confirmed their interaction through co-IP assays. Finally, to precisely map the binding regions, we used HBx truncation mutants and SREBP2-CTD/NTD plasmids. Interestingly, unlike the CTD, which interacted with all three regions (X1, X2, and X3) (**Figures 2E, F**), the NTD only bound to the 101-154 amino acid region of HBx, corresponding to X3 (**Figures 2G, H**). In conclusion, these results indicate that SREBP2 and HBx exhibit strong protein-protein interactions.

### SREBP2 inhibits HBx nuclear translocation

**Figure 3:**
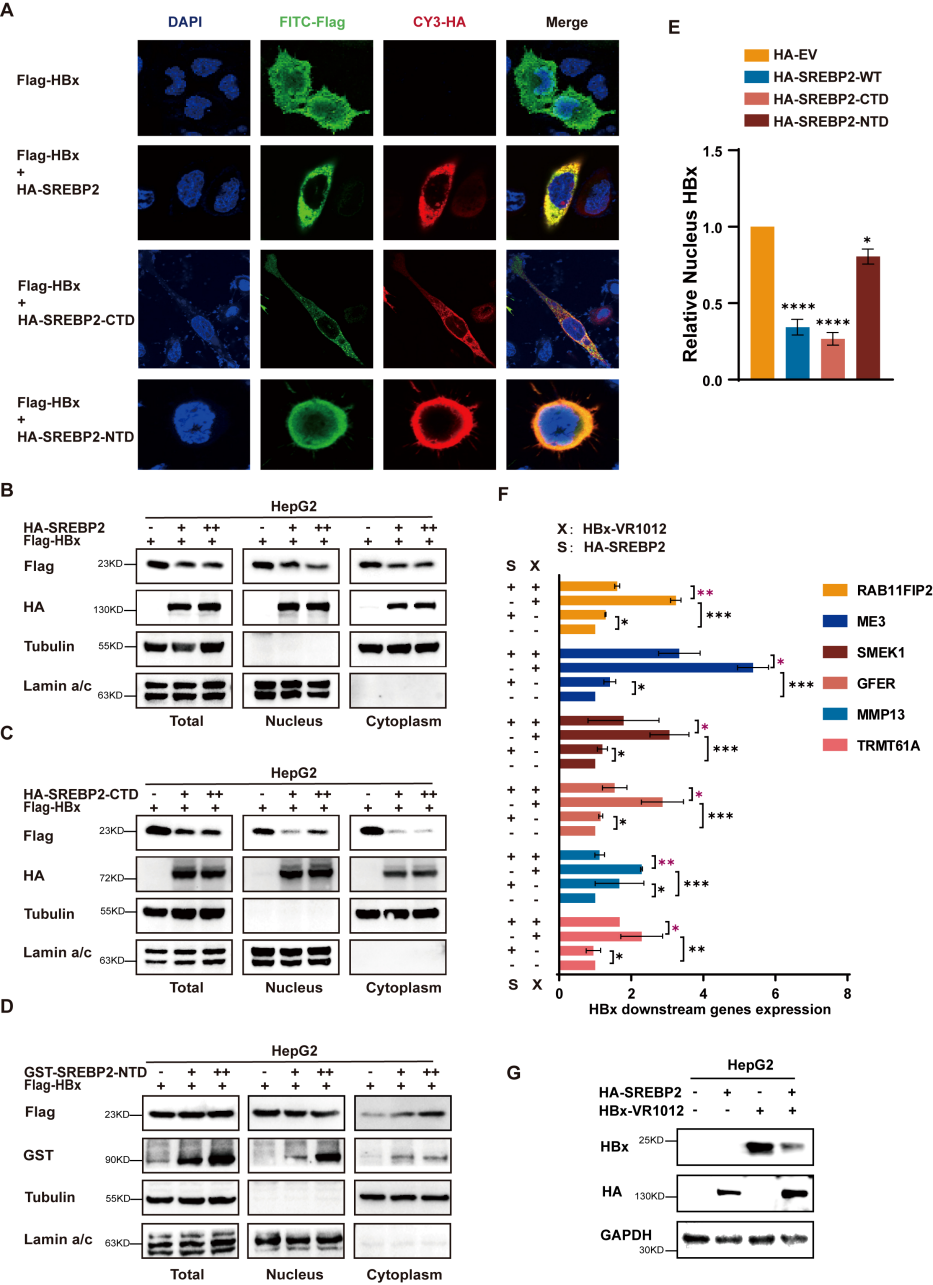
SREBP2 reduces HBx nucleus translocation. **(A)** HepG2 cells were transfected with Flag-HBx alone or co-transfected with HA-SREBP2-WT or truncated versions of HA-SREBP2. After 36 hours, cells were washed with PBS and analyzed by immunofluorescence with anti-HA and anti-Flag antibodies. **(B, C)** HepG2 cells were transfected with Flag-HBx or HA-SREBP2 (WT or CTD). After 48 hours, cells were collected in two sets: one for total protein extraction and the other for separating nuclear and cytoplasmic fractions. Immunoblotting was conducted on cell lysates using HA, Flag, Tubulin, and Lamin A/C antibodies. **(D)** HepG2 cells were transfected with Flag-HBx or GST-SREBP2-NTD. After 48 hours, cells were collected in two sets: one for total protein extraction and the other for separating nuclear and cytoplasmic fractions. Immunoblotting was conducted on cell lysates using GST, Flag, Tubulin, and Lamin A/C antibodies. **(E)** ImageJ software was used to quantify the nuclear localization of HBx in cells transfected with equal amounts of HA-SREBP2-WT, CTD, or NTD constructs. **(F)** HepG2 cells were transfected with HBx or HA-SREBP2 plasmids, harvested after 48 hours, and qPCR was performed to detect HBx downstream target genes. **(G)** Immunoblotting was conducted on cell lysates from panel **(F)** using antibodies against HA, HBx, and GAPDH. *, p≤0.05; **, p≤0.01; ***, p≤0.001; ****, p≤0.0001.

SREBP2 is known as a transcription factor located in the endoplasmic reticulum (ER) membrane, playing a role in regulating cholesterol metabolism, and the nuclear translocation of HBx is essential for regulating viral and host gene expression (5, 16, 21), leading us to hypothesize that the SREBP2-HBx interaction may influence HBx nuclear translocation, thereby inhibiting HBV replication. We co-transfected HepG2 cells with SREBP2-WT, CTD, NTD, and HBx expression plasmids. After 30 hours, the cells were examined using confocal microscopy. We observed that when HBx was transfected alone, it distributed throughout the cell. However, when co-expressed with SREBP2 WT and CTD, most HBx proteins were retained in the cytoplasm, however, not so significantly with NTD co-transfected. In summary, less HBx was observed in the nucleus when co-transfected with SREBP2 and CTD (**Figure 3A**). To confirm these results, we transfected HepG2 cells with SREBP2-WT, CTD, NTD, and Flag-HBx plasmids, then isolated total, nuclear, and cytoplasmic proteins. Western blot analysis revealed that nuclear HBx levels significantly decreased in cells co-expressing SREBP2-WT or CTD compared to HBx alone, whereas NTD expression had no noticeable impact (**Figures 3B–E**). Furthermore, quantitative PCR analysis of HBx downstream target genes revealed that SREBP2 significantly suppressed HBx-mediated gene upregulation (**Figures 3F, G**). Together, these findings demonstrate that SREBP2 impairs HBx nuclear translocation.

### SREBP2-CTD - HBx complex could be detected extracellular

**Figure 4:**
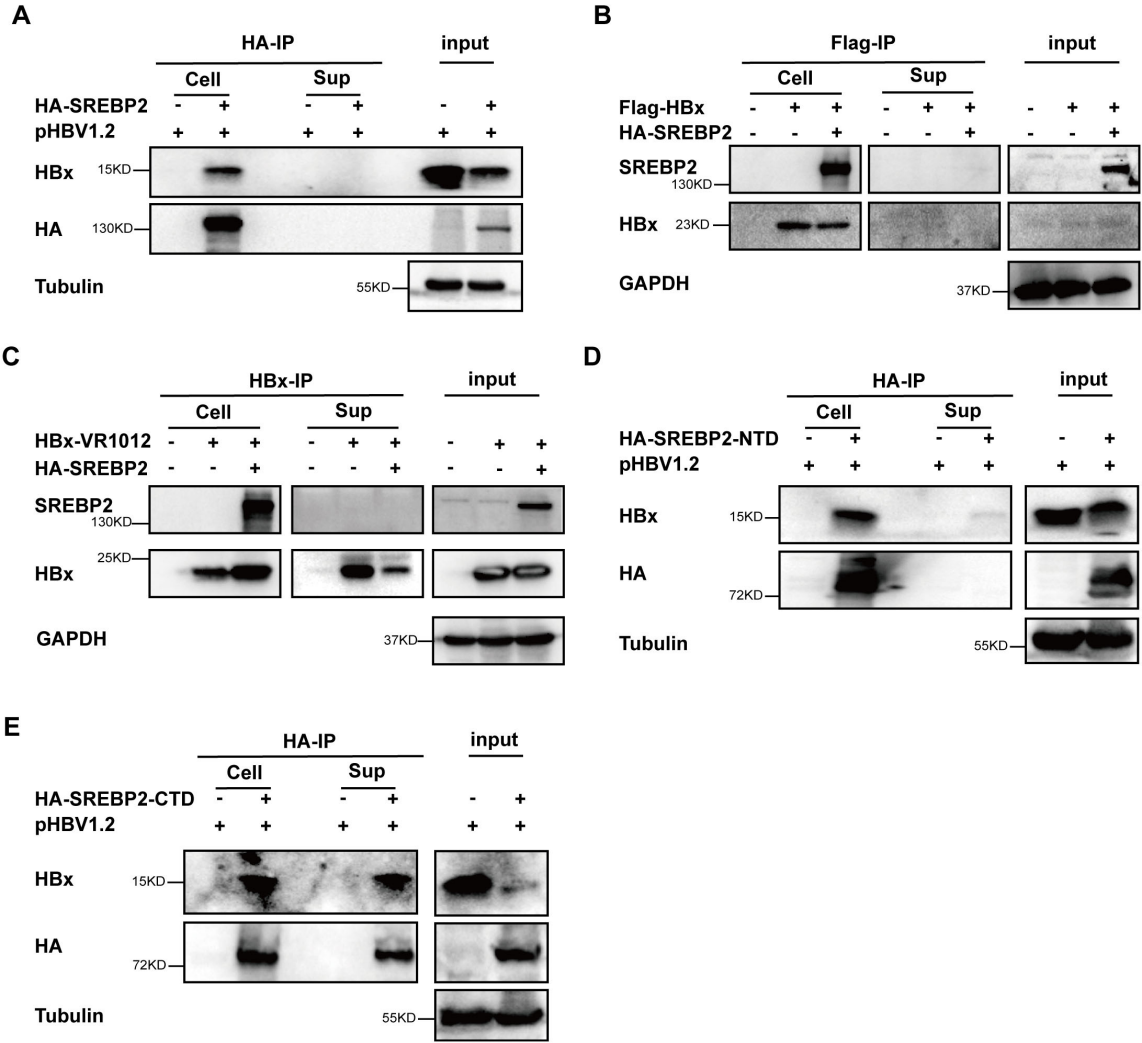
The CTD of SREBP2 is secreted extracellularly along with HBx. **(A)** HepG2 cells were transfected with pHBV1.2 alone or co-transfected with HA-SREBP2. After 28 hours, cells and supernatants were collected for co-IP with anti-HA, anti-HBx, or anti-Tubulin antibodies. **(B)** HepG2 cells were co-transfected with Flag-HBx or HA-SREBP2 plasmids. After 28 hours, cells and supernatants were collected for co-IP with anti-SREBP2, anti-HBx, or anti-GAPDH antibodies. **(C)** HepG2 cells were co-transfected with HBx-VR1012 or HA-SREBP2 plasmids. After 28 hours, cells and supernatants were collected for co-IP with anti-SREBP2, anti-HBx, or anti-GAPDH antibodies. **(D)** HepG2 cells were transfected with pHBV1.2 alone or co-transfected with HA-SREBP2-NTD. After 28 hours, cells and supernatants were collected for co-IP with anti-HA, anti-HBx, or anti-Tubulin antibodies. **(E)** HepG2 cells were transfected with pHBV1.2 alone or co-transfected with HA-SREBP2-CTD. After 28 hours, cells and supernatants were collected for co-IP with anti-HA, anti-HBx, or anti-Tubulin antibodies.

Interestingly, as we can see in **Figures 3B, C**, the total and cytoplasmic levels of HBx also decreased in the SREBP2 WT or CTD co-transfected samples. Given reports that SREBP2-CTD can be detected in the blood (22), we hypothesized that CTD may facilitate the secretion of HBx extracellularly. We co-transfected HepG2 cells with HA-SREBP2 WT and pHBV1.2 plasmids. After 30 hours, we collected the supernatants and cells and incubated them with HA beads for co-IP analysis, while HA-SREBP2 and HBx was not detected in the IP samples of supernatants (**Figure 4A**). In addition, both HA-SREBP2 and Flag-HBx could not be detected in the supernatant (**Figure 4B**). And we confirmed this result by detecting SREBP2- CTD in the supernatants along with HBx, but not WT and NTD. Interestingly, HBx could be detected in the supernatants of pHBV1.2 transfected samples, but not Flag-HBx transfected samples (**Figures 4B, C**), which indicated the Flag-tag might block the secreting of HBx protein. Interaction between SREBP2-WT, CTD, NTD, and HBx was also observed in cell lysates (**Figures 4A–E**), consistent with our previous results. Collectively, these results suggest that SREBP2-CTD can be secreted and interact with HBx extracellularly, thereby reducing the total HBx levels inside the cells, and SREBP2 might facilitate HBx secretion by its CTD.

### SREBP2 inhibits HBV replication in the absence of HBx

**Figure 5:**
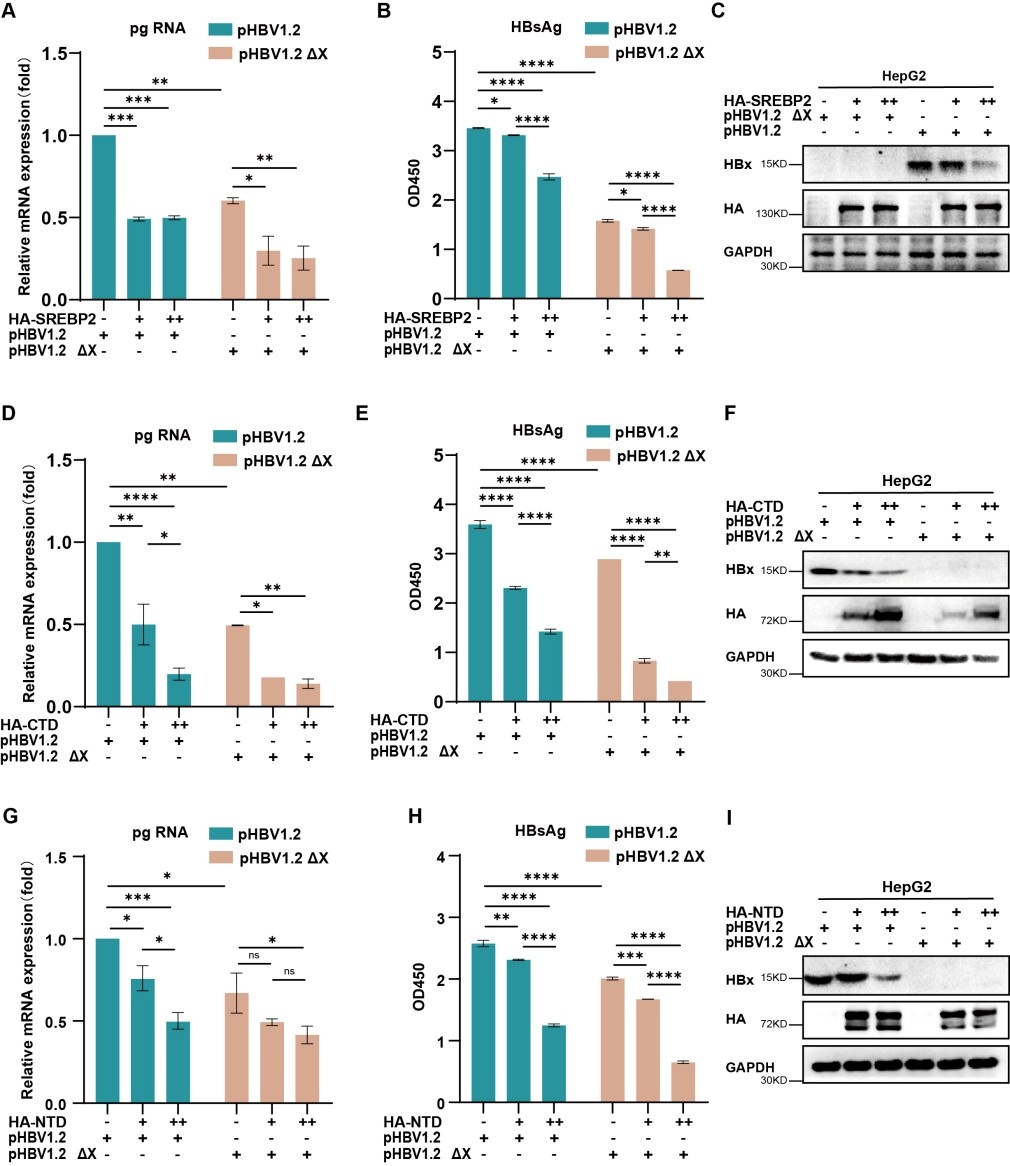
SREBP2 inhibits HBV replication through mechanisms beyond its interaction with HBx. **(A, B)** HepG2 cells were transfected with pHBV1.2 or pHBV1.2 ΔX along with HA-SREBP2. After 48 hours, cells and supernatants were harvested for qPCR or ELISA analysis. **(C)** Immunoblotting was conducted on cell lysates from panels **(A, B)** using antibodies against HBx, HA, or GAPDH. **(D, E)** HepG2 cells were transfected with pHBV1.2 or pHBV1.2 ΔX along with HA-SREBP2-CTD. After 48 hours, cells and supernatants were harvested for qPCR or ELISA analysis. **(F)** Immunoblotting was conducted on cell lysates from panels **(D, E)** using antibodies against HBx, HA, or GAPDH. **(G, H)** HepG2 cells were transfected with pHBV1.2 or pHBV1.2 ΔX along with HA-SREBP2-NTD. After 48 hours, cells and supernatants were harvested for qPCR or ELISA analysis. **(I)** Immunoblotting was conducted on cell lysates from panels **(G, H)** using antibodies against HBx, HA, or GAPDH. ns, p>0.05; *, p≤0.05; **, p≤0.01; ***, p≤0.001; ****, p≤0.0001.

To further elucidate the relationship between SREBP2 and HBV replication, we examined whether the inhibitory effects of SREBP2 were entirely dependent on HBx. Co-transfection experiments in HepG2 cells with SREBP2 wild-type (WT) or its variants, along with either pHBV1.2 or pHBV1.2ΔX plasmids, revealed that pregenomic RNA (pgRNA) and HBsAg levels were significantly suppressed even in the absence of HBx (**Figures 5A–I**). This observation suggests that SREBP2-WT and its domains (CTD and NTD) may regulate HBV replication through additional mechanisms beyond their interaction with HBx, highlighting the complexity of SREBP2-mediated viral suppression and warranting further investigation into these alternative regulatory pathways.

## Discussion

The interaction between lipid metabolism and viral replication is increasingly recognized as a crucial factor in the pathogenesis of chronic viral infections, including hepatitis B virus (HBV) (22). Cholesterol, as a fundamental lipid component, plays diverse roles in cellular homeostasis, viral entry, replication, and assembly (9, 23). HBV has been shown to manipulate the host’s lipid metabolism to facilitate its own replication, making lipid regulatory pathways attractive therapeutic targets (15).

Research has demonstrated that HBV replication is intricately linked to lipid metabolism, with viral replication often inducing alterations in lipid homeostasis to favor viral persistence (22). For instance, studies have shown that cholesterol-rich microdomains in cellular membranes, known as lipid rafts, are crucial for the assembly and release of HBV particles (22). Additionally, the modulation of key cholesterol-regulating enzymes and transporters has been implicated in HBV replication. For example, cholesterol 25-hydroxylase (CH25H), an interferon-stimulated gene involved in cholesterol metabolism, inhibits HBV replication by preventing the nuclear translocation of the HBx protein (16). This suggests that disrupting cholesterol biosynthesis or transport pathways could hinder HBV replication.

SREBP2 is a key regulator of cholesterol biosynthesis and homeostasis (24, 25), controlling genes involved in cholesterol uptake and synthesis, such as HMG-CoA reductase (HMGCR) and the LDL receptor, which may also influence HBV replication (8, 26). In this study, we observed a decrease in SREBP2 expression in PBMCs after HBV infection, likely due to extracellular viral stimuli, which may alter lipid metabolism in these cells. Importantly, as PBMCs cannot be directly infected by HBV, this reduction in SREBP2 is likely linked to immune responses. In contrast, hepatocytes show increased SREBP2 expression following HBV entry, which suppresses HBV replication. These findings suggest that SREBP2 may exert distinct functions in different cell types, and further investigation is needed to clarify the underlying mechanisms of its regulation in HBV infection.

Additionally, our study demonstrates that SREBP2 interacts directly with HBx, inhibiting its nuclear translocation and suppressing HBV replication. This highlights a novel antiviral role of SREBP2 beyond cholesterol regulation. The inhibition of HBV replication is likely due to SREBP2’s interference with HBx’s role as a transcriptional co-activator of viral genes. Furthermore, our data suggest that modulating SREBP2 activity—such as through inhibition of its maturation by agents like 25-hydroxycholesterol (25HC) or betulinic acid—may offer a new therapeutic strategy for controlling HBV infection.

The potential of SREBP2 as a therapeutic target in HBV treatment is promising, particularly because of its dual role in cholesterol regulation and viral inhibition. Agents that inhibit SREBP2 maturation, such as PF-429242 (27), which targets site-1 protease (S1P), could not only disrupt cholesterol biosynthesis but also impair HBV replication by blocking HBx’s nuclear functions. This dual targeting mechanism provides a unique advantage in developing anti-HBV therapies. Furthermore, SREBP2’s interaction with HBx suggests that pharmacologically targeting this pathway may be particularly effective in patients where HBx plays a significant role in promoting viral replication and pathogenesis, such as in those with high viral loads or those at risk for hepatocellular carcinoma (HCC).

The HBx protein plays multiple roles in HBV replication and pathogenesis (28). Beyond its known function in facilitating HBV RNA synthesis by degrading the Smc5/6 complex (7), HBx interacts with numerous host proteins to manipulate cellular processes for viral advantage (29). For instance, HBx has been shown to modulate apoptosis, cell proliferation, and immune evasion, contributing to the persistence of HBV infection and the progression to HCC (5). SREBP2, especially its C-terminal domain (CTD), interacts with HBx to reduce its intracellular levels and may aid in its extracellular secretion, underscoring a regulatory role for SREBP2 in HBx dynamics. Notably, while SREBP2-CTD can be secreted and detected in the extracellular environment along with HBx, the presence of a Flag tag on HBx seems to inhibit HBx secretion (**Figures 4B, C**), likely due to structural incompatibility with the secretion pathway. These findings reveal new insights into SREBP2’s involvement in HBV replication and the potential mechanisms of HBx secretion regulation. Targeting these interactions could provide novel avenues for therapeutic intervention, potentially disrupting the virus’s ability to hijack host cellular machinery.

**Figure 6:**
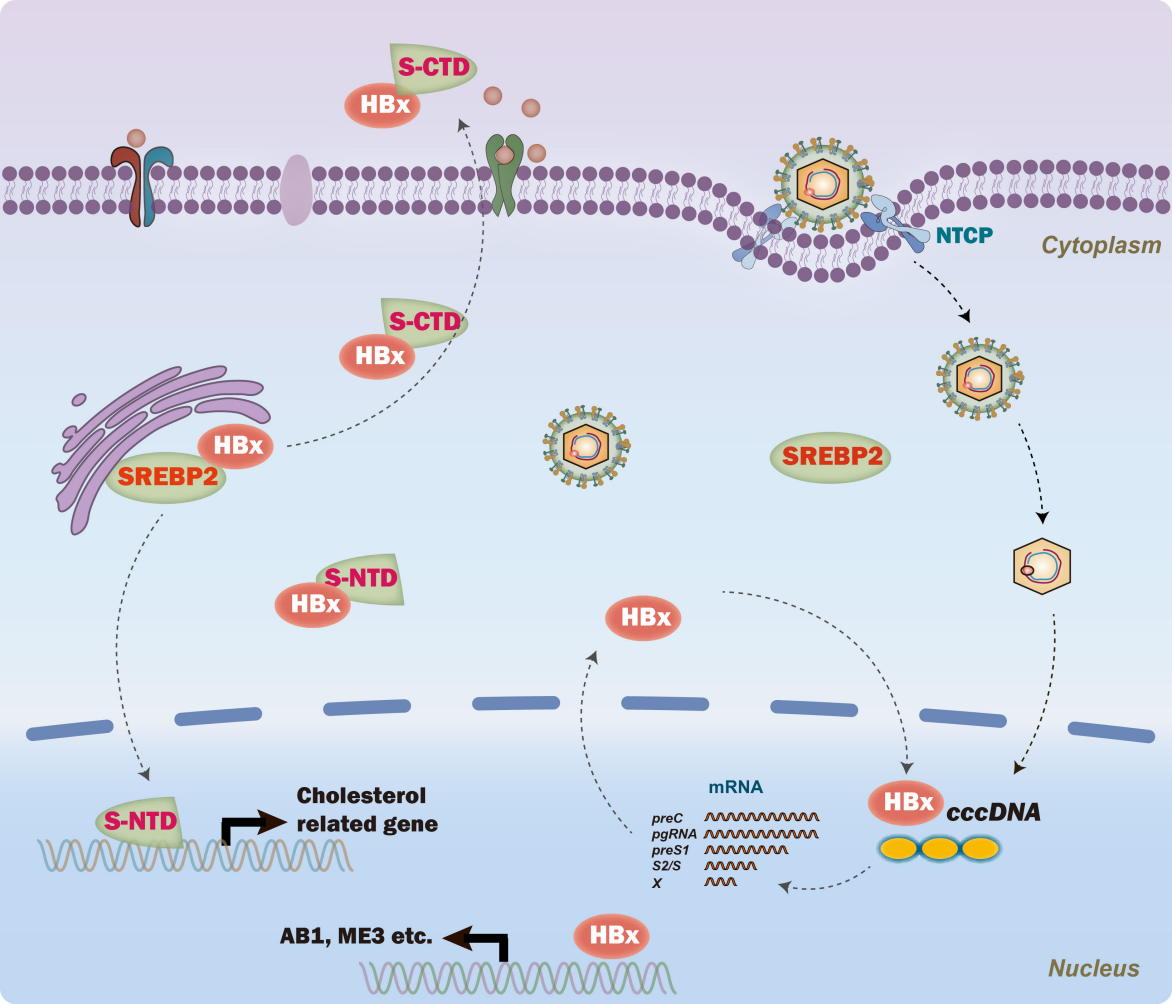
A proposed model of SREBP2’s role in regulating hepatitis B virus (HBV) replication. Briefly, SREBP2 interacts with HBx, inhibiting its nuclear translocation and thereby reducing HBV transcription from the cccDNA template, independent of its N-terminal domain (NTD). In parallel, the C-terminal domain (CTD) of SREBP2 interacts with HBx to facilitate cellular export, further limiting HBx nuclear translocation.

Based on our findings, it was proposed that SREBP2 regulated HBV replication by inhibiting HBx nucleus translocation (**Figure 6**). The identification of the SREBP2-HBx interaction presents promising avenues for exploring the role of lipid metabolism in viral replication. Future studies should focus on unraveling the precise molecular mechanisms by which SREBP2 regulates HBx activity, potentially providing new insights into therapeutic strategies. Investigating whether other SREBP family members, such as SREBP1, similarly impact HBV replication could also enhance our understanding of lipid metabolism’s role in HBV pathogenesis. Given HBx’s pivotal role in both viral replication and disease progression, targeting the SREBP2-HBx axis could introduce a novel class of anti-HBV therapies that combine metabolic and antiviral actions. Additionally, our findings reveal that SREBP2 regulates HBV replication through mechanisms beyond its interaction with HBx, highlighting the importance of investigating these alternative pathways to fully understand the complex host-virus dynamics.

## Data Availability

The datasets presented in this study can be found in online repositories. The names of the repository/repositories and accession number(s) can be found in the article/**Supplementary Material**.

## Supplementary material

Supplementary Figure 1
**(A)** HepG2 cells were transfected with pHBV1.2 or co-transfected with HA-tagged SREBP2 expression plasmids, followed by treatment with 25-HC. After 48 hours, cells were harvested for qPCR (HBV DNA) and Elisa analysis (HBeAg). **(B)** Cytotoxicity in HepG2 cells treated with 10 mM betulin, 50 mM PF-429242, 10 mM 25-HC, or 50 mM cholesterol was measured.
