# Supplementary figures and images for: Cholesterol metabolism regulator SREBP2 inhibits HBV replication via suppression of HBx nuclear translocation
Source: Front Immunol. 2025 Jan 13;15:1519639. doi: 10.3389/fimmu.2024.1519639 (PMC11769810; doi:10.3389/fimmu.2024.1519639)

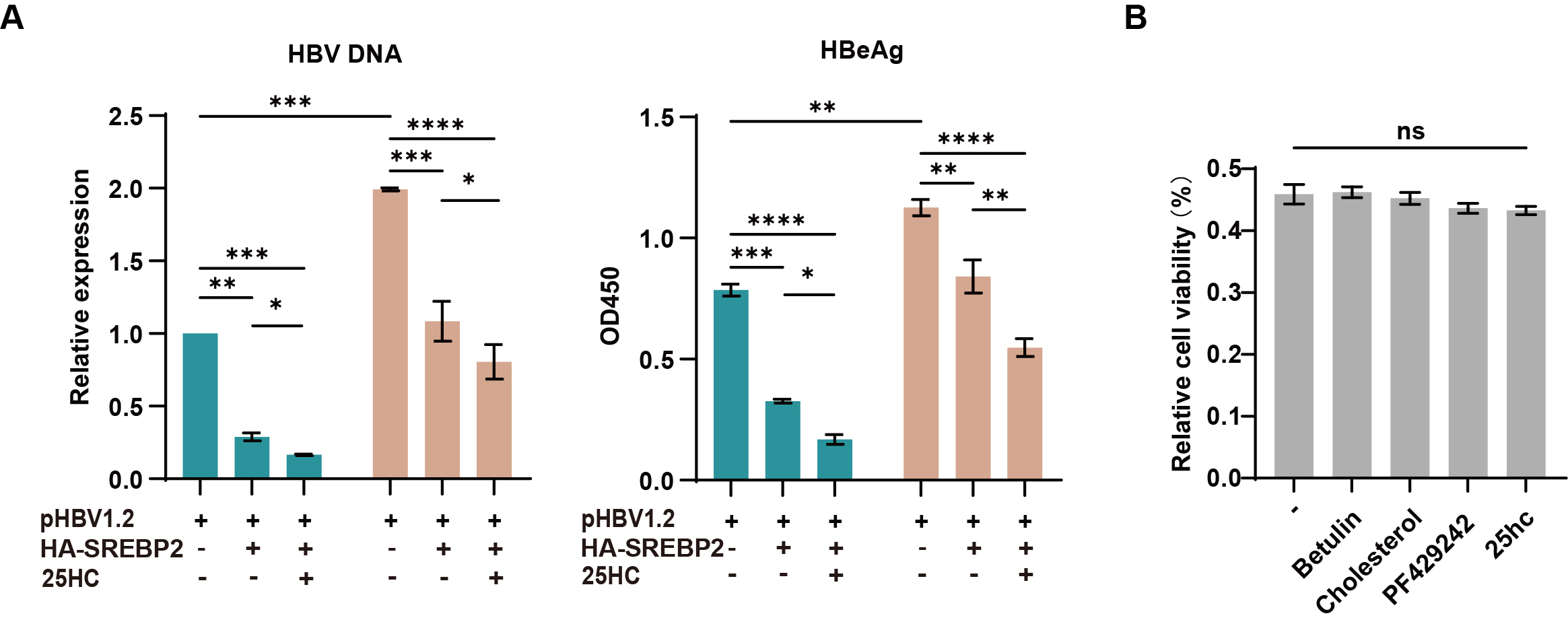

Supplement: Supplementary Figure 1 — (A) HepG2 cells were transfected with pHBV1.2 or co-transfected with HA-tagged SREBP2 expression plasmids, followed by treatment with 25-HC. After 48 hours, cells were harvested for qPCR (HBV DNA) and Elisa analysis (HBeAg). (B) Cytotoxicity in HepG2 cells treated with 10 mM betulin, 50 mM PF-429242, 10 mM 25-HC, or 50 mM cholesterol was measured. [file Image1.jpeg]
