# Supplementary material for: Cholesterol metabolism regulator SREBP2 inhibits HBV replication via suppression of HBx nuclear translocation
Source: Front Immunol. 2025 Jan 13;15:1519639. doi: 10.3389/fimmu.2024.1519639 (PMC11769810; doi:10.3389/fimmu.2024.1519639)
Supplement: Supplementary file 2 [file Table1.docx]

**Table S1. Clinical characteristics of HBV-infected patients**

|  |  |  | **HBV-infected patients** | | | | |
| --- | --- | --- | --- | --- | --- | --- | --- |
| No. | Sex | Age (years) | AST (U/L) | ALT (U/L) | HBV DNA (copies/mL) | HBsAg (IU/mL) | HBeAg (S/CO) |
| 1 | M | 70 | 130.6 | 185.3 | 55300 | >250 | 0.42 |
| 2 | F | 46 | 57 | 56.9 | 355000 | 49813.76 | 1052 |
| 3 | M | 33 | 33.1 | 76.2 | 33700 | / | / |
| 4 | M | 42 | 27 | 34.7 | 244000 | / | / |
| 5 | M | 43 | 119.7 | 116 | 83500000 | 4978.35 | 984.091 |
| 6 | M | 56 | 25.6 | 20.4 | 282 | 1342.31 | / |
| 7 | F | 39 | 54.3 | 92.2 | 3580 | 23128.46 | / |
| 8 | F | 51 | 20.8 | 16.7 | 276 | 16008.67 | / |
| 9 | M | 57 | 48 | 62.5 | 63.6 | 30.14 | / |
| 10 | F | 39 | 18.3 | 21.5 | 170000000 | 67932.55 | 1805.888 |
| 11 | M | 64 | 53.1 | 33.3 | 126 | >250 | 0.351 |
| 12 | F | 33 | 40.7 | 62.3 | 102 | 72.9 | 0.312 |
| 13 | M | 40 | 52.6 | 101.8 | 2620 | 2477.24 | 0.342 |
| 14 | F | 26 | 36 | 46.7 | 294 | 25078.67 | / |
| 15 | F | 46 | 228.6 | 347.5 | 21200000 | >250 | 1.741 |
| 16 | F | 41 | 29.2 | 32.6 | 118000 | 2065.64 | / |
| 17 | M | 57 | 252.1 | 63.6 | 8420 | 40.55 | / |
| 18 | F | 50 | 18.2 | 14.8 | 740 | >250 | 0.451 |
| 19 | F | 29 | 17.1 | 20.9 | 1380 | 343.04 | / |
| 20 | M | 22 | / | / | 121000000 | 79262.71 | / |
| 21 | F | 49 | 14.9 | 3.5 | 9560 | >250 | 0.118 |
| 22 | F | 20 | 71 | 88 | 27000000 | 12951.13 | / |
| 23 | M | 35 | 62 | 45.8 | 69.8 | 4088.35 | / |
| 24 | F | 42 | 684.3 | 534.3 | 7010000 | / | / |
| 25 | F | 50 | 34 | 32.9 | 3580 | 3117.27 | / |
| 26 | M | 42 | 91.2 | 127 | 375 | 1414.16 | / |
| 27 | F | 63 | 55 | 33.5 | 114000000 | >250 | 915.267 |
| 28 | F | 47 | 31.2 | 19.2 | 879 | 2655.45 | 0.38 |
| 29 | M | 55 | 61.1 | 54.4 | 1970 | / | / |
| 30 | M | 52 | 41.4 | 52.3 | 305000 | 8515.93 | 1.492 |
| 31 | M | 36 | 35.4 | 42.9 | 25400 | 10965.84 | / |
| 32 | M | 37 | 30.7 | 37.6 | 439000000 | 72226.72 | 1480.39 |
| 33 | M | 50 | 98.8 | 151.3 | 2180 | 342.4 | 0.451 |
| 34 | M | 51 | 42.8 | 48.7 | 1280000 | 3736.25 | 23.547 |
| 35 | F | 57 | 25.1 | 20.2 | 173000 | 8549.05 | / |
| 36 | M | 24 | 18.2 | 16.9 | 332000000 | >250 | 1567.955 |
| 37 | M | 57 | 184 | 130 | 505 | >250 | 824.993 |
| 38 | M | 34 | 59.9 | 118.8 | 119000000 | 23885.9 | / |
| 39 | F | 41 | 26.4 | 21.6 | 12000 | 11650.86 | / |
| 40 | M | 24 | 29.7 | 43 | 62700000 | 71298.23 | / |
| 41 | F | 40 | 53.1 | 53.4 | 346000 | 5775.62 | 0.418 |
| 42 | M | 51 | / | / | 1280000 | 383.13 | / |
| 43 | M | 62 | / | / | 137000 | 1453.17 | / |
| 44 | M | 38 | 75.6 | 184.1 | 140000 | 6222.78 | / |

**Table S2. Clinical characteristics of healthy controls**

|  |  |  | **Healthy controls** | |
| --- | --- | --- | --- | --- |
| NO | Sex | Age/year | AST (U/L) | ALT (U/L) |
| 1 | M | 51 | 35.1 | 46.4 |
| 2 | F | 56 | 46.1 | 59.6 |
| 3 | M |  | 26.2 | 18.4 |
| 4 | M | 52 | 19.3 | 19.4 |
| 5 | F | 34 | // |  |
| 6 | F | 34 | / | / |
| 7 | M | 69 | / | / |
| 8 | F | 45 | / | / |
| 9 | M | 43 | / | / |
| 10 | F | 51 | / | / |
| 11 | F | 32 | / | / |
| 12 | M | 18 | / | / |
| 13 | M | 31 | / | / |
| 14 | F | 46 | / | / |
| 15 | M | 27 | / | / |
| 16 | M | 18 | / | / |
| 17 | F | 26 | / | / |
| 18 | F | 45 | / | / |

**Table S3: Primers for infusion cloning**

**SREBP2**

| Primers | | Sequence |
| --- | --- | --- |
| WT | Forward | 5'-TCGCGGCCGCTCTAGAATGGACGACAGCGGCGAGCT-3' |
| WT | Reverse | 5'-AGGCGCCTGGTCTAGATCAGGAGGCGGCAATGGCAG-3' |
| CTD | Forward | 5'-TCGCGGCCGCTCTAGA ATGTCACGGATTCTTCTGTGT-3' |
| NTD | Forward | 5'-AGGCGCCTGGTCTAGA TTAGCGGTCTACCATGCCCAG-3' |

**HBx**

| Primers | | Sequence |
| --- | --- | --- |
| WT | Forward | 5'-TCGCGGCCGCTCTAGAATGGCTGCTCGGGTGTGCTGC-3' |
| WT | Reverse | 5'-AGGCGCCTGGTCTAGATTAGGCAGAGGTGAAAAAG-3' |
| X1 | Forward | 5'-TCGCGGCCGCTCTAGAATGGCTGCTCGGGTGTGCTGC-3' |
| X1 | Reverse | 5'-AGGCGCCTGGTCTAGATTACCCGTGGTCGGCCGGAAC-3' |
| X2 | Forward | 5'-TCGCGGCCGCTCTAGAATGGCGCACCTCTCTTTAC-3' |
| X2 | Reverse | 5'-AGGCGCCTGGTCTAGATTAGAGTCCAAGAGTCCTC-3' |
| X3 | Forward | 5'-TCGCGGCCGCTCTAGAATGTCAGCAATGTCAACGACC-3' |
| X3 | Reverse | 5'-AGGCGCCTGGTCTAGATTATTAGGCAGAGGTGAAAAA-3' |

**Table S4: Primers sequences for quantitative real-time PCR**

| Primers | | Sequence |
| --- | --- | --- |
| GAPDH | Forward | 5'-CGGATTTGGTCGTATTGGG-3' |
| GAPDH | Reverse | 5'-TCTCGCTCCTGGAAGATGG-3' |
| SREBP2 | Forward | 5'-AACGGTCATTCACCCAGGTC-3' |
| SREBP2 | Reverse | 5'-GGCTGAAGAATAGGAGTTGCC-3' |
| HBV DNA | Forward | 5'-GAGTGTGGATTCGCACTCC-3' |
| HBV DNA | Reverse | 5'-GAGGCGAGGGAGTTCTTCT-3' |
| HBV pgRNA | Forward | 5'-TCTTGCCTTACTTTTGGAAG-3' |
| HBV pgRNA | Reverse | 5'-AGTTCTTCTTCTAGGGGACC-3' |
| HMGCR | Forward | 5'-TGATTGACCTTTCCAGAGCAAG-3' |
| HMGCR | Reverse | 5'-CTAAAATTGCCATTCCACGAGC-3' |
| LDLR | Forward | 5'-TCTGCAACATGGCTAGAGACT-3' |
| LDLR | Reverse | 5'-TCCAAGCATTCGTTGGTCCC-3' |
| ME3 | Forward | 5'-TGAAGAAGCGCGGATACGATG-3' |
| ME3 | Reverse | 5'-GAAAGCAGGGCGGGATTAGG-3' |
| SMEK1 | Forward | 5'-CCTTGGCATGGATGATACAC-3' |
| SMEK1 | Reverse | 5'-CTCCTGCATGACAAACTCTC-3' |
| GFER | Forward | 5'-AGCGGGACACCAAGTTTAGG-3' |
| GFER | Reverse | 5'-GCCATGTCTTGCTGCTGTTC-3' |
| TRMT61A | Forward | 5'-GAGGTGGCTGGGTGTATGTG-3' |
| TRMT61A | Reverse | 5'-GGTGATGAGGGCGATGTCTG-3' |
| RAB11FIP2 | Forward | 5'-CATGCCCGATGCCAATAGTG-3' |
| RAB11FIP2 | Reverse | 5'-GTGCCAGCCTTCAGTTTCTC-3' |
| MMP13 | Forward | 5'-ACTGAGAGGCTCCGAGAAATG-3' |
| mmp13 | Reverse | 5'-GAACCCCGCATCTTGGCTT-3' |

**Table S5: SgRNAs and knockout sequencing primers for SREBP2**

| Primers | | Sequence |
| --- | --- | --- |
| SREBP2 | Forward | 5'-CACC GTGAATGACCGTTGCACTGA-3' |
| SREBP2 | Reverse | 5'-AAAC TCAGTGCAACGGTCATTCAC-3' |
| SREBP2 KO seq | Forward | GTTTCCTGACCCATTTCTGC |
| SREBP2 KO seq | Reverse | TCATTACCGTCTGTTGTTGC |
